# Dysregulated calcium homeostasis prevents plasma membrane repair in Anoctamin 5/TMEM16E-deficient patient muscle cells

**DOI:** 10.1038/s41420-019-0197-z

**Published:** 2019-07-18

**Authors:** Goutam Chandra, Aurelia Defour, Kamel Mamchoui, Kalpana Pandey, Soumya Mishra, Vincent Mouly, SenChandra Sreetama, Mohammad Mahad Ahmad, Ibrahim Mahjneh, Hiroki Morizono, Nagarajan Pattabiraman, Anant K. Menon, Jyoti K. Jaiswal

**Affiliations:** 1 Center of Genetic Medicine Research, Children’s National Health System, 111 Michigan Avenue, NW, Washington, DC 20010 USA; 2 Center for Research in Myology, Sorbonne Universités, UPMC Université Paris 06, INSERM UMRS974, 47 Boulevard de l’hôpital, 75013 Paris, France; 3 Department of Biochemistry, Weill Cornell Medical College, New York, NY 10065 USA; 4 Department of Neurology, MRC Oulu, Oulu University Hospital and University of Oulu, Oulu, Finland; 5 Department of Genomics and Precision Medicine, George Washington University, Washington, DC 20037 USA; 6 MolBox LLC, 8115 Fenton Street #304, Silver Spring, Maryland 20910 USA; 7 Present Address: Aix Marseille Université, UMR_S 910, Génétique Médicale et Génomique Fonctionnelle, 13385 Marseille, France

**Keywords:** Neuromuscular disease, Mechanisms of disease

## Abstract

Autosomal recessive mutations in Anoctamin 5 (*ANO5/TMEM16E*), a member of the transmembrane 16 (TMEM16) family of Ca^2+^-activated ion channels and phospholipid scramblases, cause adult-onset muscular dystrophies (limb girdle muscular dystrophy 2L (LGMD2L) and Miyoshi Muscular Dystrophy (MMD3). However, the molecular role of ANO5 is unclear and *ANO5* knockout mouse models show conflicting requirements of ANO5 in muscle. To study the role of ANO5 in human muscle cells we generated a myoblast line from a MMD3-patient carrying the c.2272C>T mutation, which we find causes the mutant protein to be degraded. The patient myoblasts exhibit normal myogenesis, but are compromised in their plasma membrane repair (PMR) ability. The repair deficit is linked to the poor ability of the endoplasmic reticulum (ER) to clear cytosolic Ca^2+^ increase caused by focal plasma membrane injury. Expression of wild-type ANO5 or pharmacological prevention of injury-triggered cytosolic Ca^2+^ overload enable injured patient muscle cells to repair. A homology model of ANO5 shows that several of the known LGMD2L/MMD3 patient mutations line the transmembrane region of the protein implicated in its channel activity. These results point to a role of cytosolic Ca^2+^ homeostasis in PMR, indicate a role for ANO5 in ER-mediated cytosolic Ca^2+^ uptake and identify normalization of cytosolic Ca^2+^ homeostasis as a potential therapeutic approach to treat muscular dystrophies caused by ANO5 deficit.

## Introduction

Mutations in the *Anoctamin 5* (*ANO5)/ TransMEMbrane 16E (TMEM16E)* gene underlie the most common adult onset recessive muscular dystrophy in Northern Europe^1–3^. This includes limb-girdle muscular dystrophy type 2 L (LGMD 2 L) and Miyoshi myopathy type 3 (MMD3), which are characterized by high levels of serum creatine kinase (CK) indicating myofiber damage, exercise-induced muscle pain, and rare presentation of cardiomyopathy or respiratory failure. LGMD 2 L causes proximal, asymmetric weakness of quadriceps, femoris and biceps brachii, and minimal weakness of distal lower limbs. MMD3 results in weakness of the calf and other distal muscles, making it difficult for patients to walk on their toes, which progresses and results in asymmetric weakness of proximal limb-girdle muscles^4^. *ANO5/TMEM16E* gene mutations also result in the autosomal dominant bone disorder gnathodiaphyseal dysplasia (GDD). Thus, another name for *ANO5* is *GDD1*^5^. ANO5/TMEM16E/GDD1 (henceforth ANO5) belongs to the ten-member human Anoctamin/TMEM16 membrane protein family that includes Ca^2+^-activated ion channels (ANO1/TMEM16A, ANO2/TMEM16B) and phospholipid scramblases (ANO3, ANO4, ANO6, ANO7, and ANO10)^6,7^. In reconstitution-based assays ANO6/TMEM16F and its fungal homologues *Aspergillus fumigatus* TMEM16 (afTMEM16) and *Nectria haematococca* TMEM16 (nhTMEM16) show both scramblase and relatively unselective ion channel activity that are regulated by Ca^2+^ ^8–16^.

ANO5 is expressed primarily in muscle and bone, localizing to the endoplasmic reticulum (ER)^17–19^. Evidence exists in favor and against its ability to execute canonical functions of the Anoctamin/TMEM16 family, i.e. to act as a Ca^2+^-activated Cl^−^ channel and/or a phospholipid scramblase^19–25^. It is likely that similar to ANO6/TMEM16F, ANO5 is also a dual function ion channel and phospholipid scramblase. Thus, transplantation of a short stretch of amino acids (the ‘scrambling domain’ or SCRD) from ANO5 onto ANO1/TMEM16A, imparts Ca^2+^-activated phospholipid scramblase activity to this Cl^−^ channel^21,26^. Heterologous overexpression of ANO5 in the plasma membrane of HEK293 cells associates with Ca^2+^-dependent phospholipid scrambling and non-selective ion currents^22,26^.

The physiological role of ANO5 remains elusive, despite the large number of known disease-associated *ANO5* mutations^27^. In mouse, ANO5 is implicated in muscle differentiation and membrane repair^17,28^, while our studies with MMD3 patient fibroblasts revealed a deficiency in PMR ability which correlated with the patient’s clinical symptoms including elevated serum CK level, and muscle weakness and pain^29^. Consistent with this, electron microscopic analysis of patient muscle showed sarcolemmal disruptions^4,30^. Thus, Ca^2+^-dependent PMR in human muscle cells may require ANO5. Such a role has been established for dysferlin protein, the lack of which causes distinct dystrophies termed LGMD2B and Miyoshi myopathy^31,32^. However, unlike dysferlin-deficient patient muscle cells, where PMR is compromised by poor lysosomal exocytosis^33^, ANO5-deficient patient fibroblasts displayed normal injury-triggered lysosomal exocytosis^29^. Furthermore, ANO5 overexpression did not rescue the muscle pathology of dysferlin-deficient mice^34^. Thus, the basis of defective PMR in ANO5-deficient human fibroblasts is distinct from that caused by dysferlin deficiency.

Pathologies noted in ANO5-deficient patients have not been uniformly recapitulated in ANO5 knockout animal models^19,28,35^. For example, muscle pathology and high serum CK, with in vivo and in vitro deficits in myogenic differentiation and ex vivo PMR deficit were noted in only one of several described ANO5 knockout mouse models^19,28,35^, whereas a recently described ANO5 knockout rabbit model demonstrated progressive muscle histological damage^36^. Thus, genetic background, disease model-specific differences and knockout strategy may affect disease manifestation. These conflicting data highlight the need to examine the effect of *ANO5* mutations directly by using patient muscle cell-based models. We now report the first patient-derived myoblast cell model for ANO5 deficit. We find that ANO5 is dispensable for myogenic differentiation of the human muscle cells, but is required for the repair of injured muscle cells by facilitating cytosolic Ca^2+^ homeostasis.

## Results

### ANO5 patient muscle cells grow and differentiate normally

**Fig. 1:**
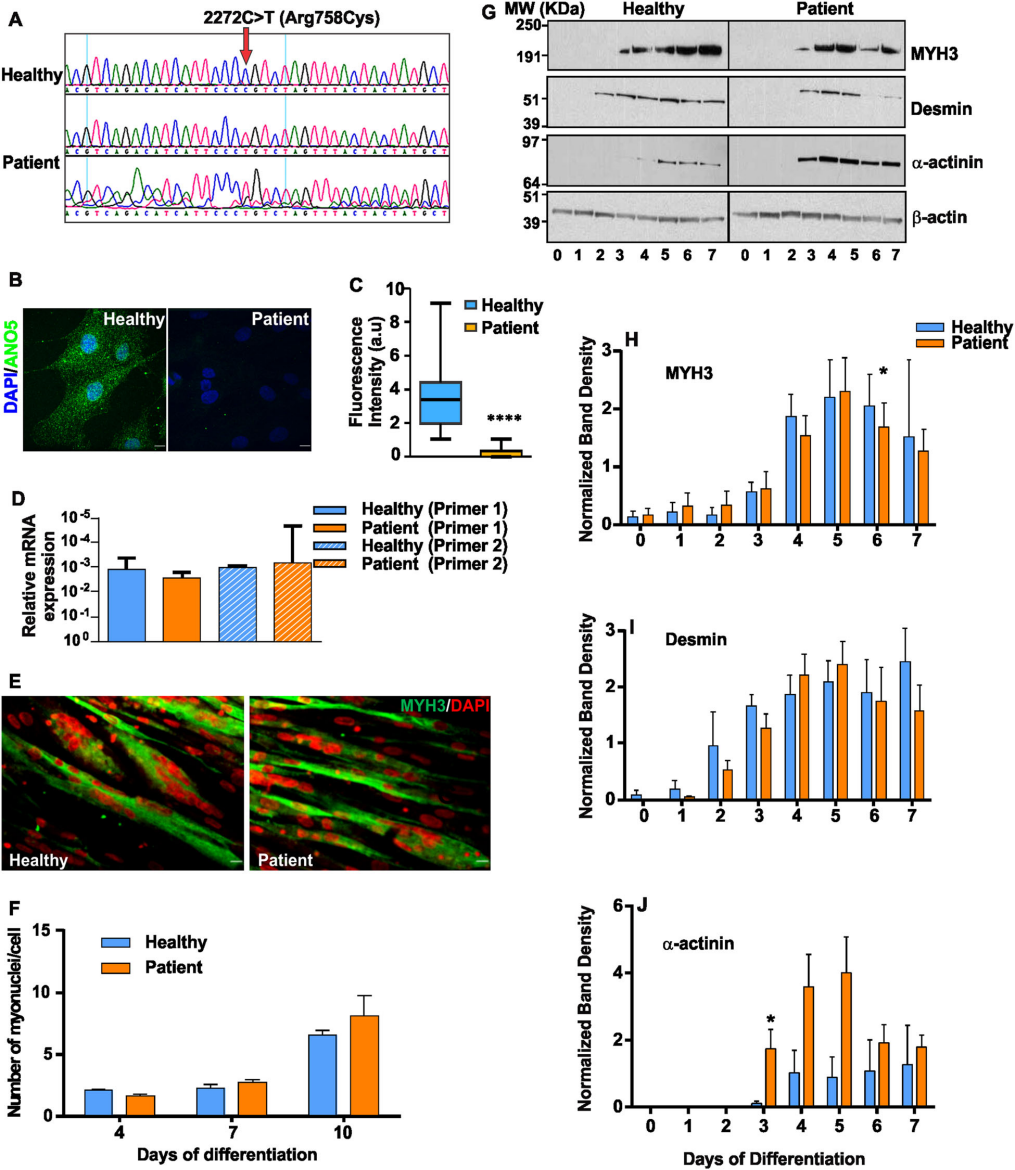
ANO5 deficit does not slow growth and differentiation of patient myoblasts. **a** Chromatogram for the sequence of the genomic DNA region of patient (middle and bottom) and healthy (top) myoblasts examining the mutation of the *ANO5* gene. **b** Images showing immunostaining and (**c**) Quantification of the fluorescence intensity of ANO5 immunostaining in healthy (*n* = 25 cells) and patient myoblasts (*n* = 41 cells, ****p < 0.0001). Scale bar = 10 µm. **d** RT PCR quantification of ANO5 transcript using two independent ANO5 mRNA-specific primers. **e** Images showing myosin heavy chain 3 (MYH3, Green) and DAPI (nuclear, Red) staining of myoblasts differentiated for 10 days. Scale bar = 10 µm. **f** Quantification of the number of myonuclei in healthy and patient myoblasts at indicated days following start of differentiation. **g**–**j** Proteins isolated from differentiating myoblasts at indicated days post differentiation are analyzed by Western Blot for differentiation markers. **g** Representative Blots and **h**–**j** Quantification of band intensity for proteins induced during differentiation **h** Myosin heavy chain 3 (MYH3), **i** Desmin and **j** α-actinin normalized to the loading control (β-actin) (*n* = 3 independent blots). The β-actin normalized band intensities were not significantly different between healthy and patient myoblasts based on the unpaired multiple *t*-tests except where indicated by *. (*p = 0.046 for **h** and *p = 0.049 for **j**

PMR and myogenesis are cellular deficits reported in LGMD2L patient and animal models^28,29,36^. To understand the basis for this pathology, we generated a stable myoblast cell line from a MMD3 patient carrying a homozygous mutation (c.2272C>T) in the *ANO5* gene (Fig. 1a), and assessed the growth and differentiation ability of these cells. Growth of patient and healthy myoblasts was indistinguishable (doubling time 18.1 ± 1.1 h vs 16.9 ± 1.1 h, respectively). Immunostaining showed significantly reduced levels of ANO5 in the patient myoblasts compared with healthy cells (Fig. 1b, c). Quantitative RT-PCR showed that ANO5 mRNA levels were unaffected (Fig. 1d), suggesting that the c.2272C>T mutation (replacement of Arginine 758 with Cysteine) causes degradation of the ANO5 protein.

Due to previous reports of decreased myogenic differentiation between the cultured WT and ANO5 deficient mouse myoblasts, we examined the ability of the patient myoblasts to undergo differentiation by allowing confluent cultures of myoblasts to differentiate for 10 days. Extent of myoblast fusion, monitored by counting myonuclei in myosin heavy chain 3 (MYH3)-positive myotubes, was similar in patient and healthy myotubes (Fig. 1e, f). Western Blot analysis of the differentiation marker proteins - MYH3, desmin, and α-actinin identified that while the expression levels of these proteins showed minor variations between healthy and patient myoblasts, the overall level and timing of induction of these markers was not reduced in the patient myoblasts (Fig. 1g–j). Above findings showed that unlike the ANO5 deficient mouse cells, MMD3 patient myoblasts do not exhibit any detectable deficit in their growth and differentiation.

### ANO5-deficient patient myoblasts show poor plasma membrane repair

**Fig. 2:**
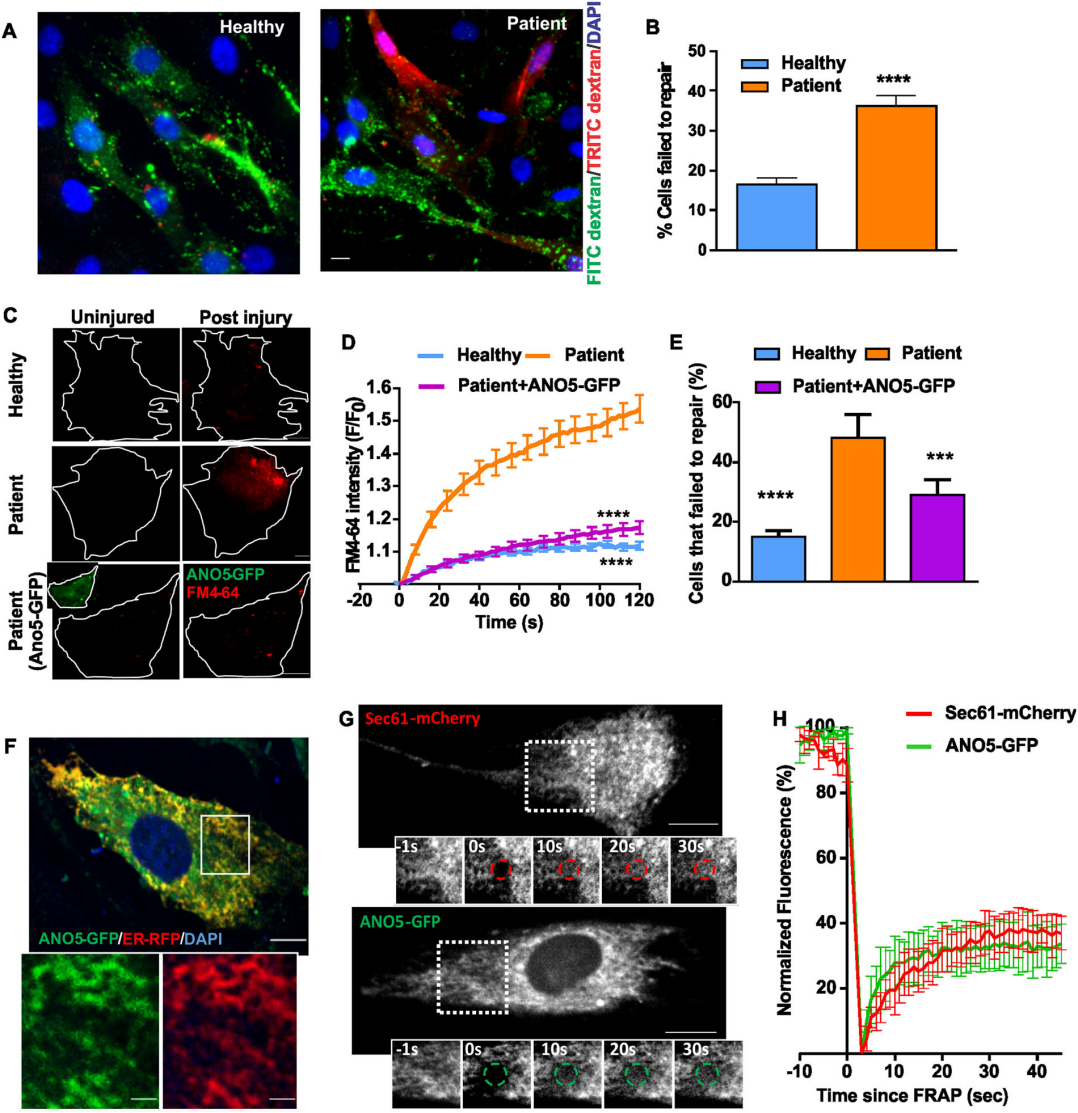
ANO5 is an ER protein that is required for muscle PMR. **a**, **b** Glass bead injury assay for healthy and patient myoblasts. **a** Representative images showing injured myoblasts (green) and cells failed to repair (green and red co-labelled myoblasts), and **b** Plot showing quantification of the cells that failed to repair (*n* = 150 cells for each; ****p < 0.0001). **c**–**e** FM1-43 dye-based assay to quantify the repair kinetics and ability of the healthy and patient myoblasts to undergo repair from focal laser injury. **c** Representative images of uninjured and injured healthy, ANO5 patient myoblasts and ANO5 patient myoblasts expressing ANO5-GFP prior to and 30 s after laser injury. **d** Kinetics of FM4-64 dye entry into the cells following laser injury *n* = 47 healthy, 37 patient myoblasts and 37 patient myoblasts transfected with ANO5-GFP (****p < 0.0001). **e** Fraction of myoblasts that repaired following laser injury in different groups., ****p < 0.0001, ***p = 0.0003. **f** Image of a healthy human myoblast co-expressing ANO5-GFP and RFP-KDEL. Inset shows a region (marked by the white box). Scale bar = 10 µm. **g,** **h** FRAP assay to monitor the ability of GFP-tagged ANO5 and the mCherry-tagged ER resident membrane protein (Sec61) to diffuse in the ER membrane. **g** Representative images showing the cell and the photobleached regions where the fluorescence recovery was monitored, and **h** plots showing the kinetics of fluorescence recovery (mean ± SEM; *n* ≥ 3 cells each)

Analysis of fibroblasts from MMD3 patients with the c.2272C>T/R758C mutation reveal that they also lack ANO5 protein (Supplemental Fig. 1a, b), and, as we reported previously^29^, they show poor PMR following focal injury (Supplemental Fig. 1c–e) or laser injury (Supplemental Fig. 1f, g). We therefore examined if myoblasts from our patient have a reduced ability to repair their plasma membrane after injury. First, we used a glass bead injury assay^33,37^ – myoblasts injured by rolling glass-beads are labeled with green fluorescein-dextran; subsequent labeling with red rhodamine dextran results in predominantly red labeling of injured cells that fail to repair. A significantly greater number of glass bead-injured patient cells were labeled red as compared to the corresponding healthy myoblasts (Fig. 2a, b). Thus, ANO5 deficit also causes poor repair of patient muscle cells. As an independent test of membrane repair ability, we used a laser injury assay^33,37^, where injury causes the lipophilic FM dye to enter the cell until the entry is prevented within a minute by PMR. In patient myoblasts, FM-dye entry continued beyond 2min post-injury (Fig. 2c–e, Supplemental Fig. 2a (trace labeled ‘Patient’)), indicating poor PMR.

We next tested if ANO5 expression could rescue the PMR deficit of patient cells. We used transient transfection to express ANO5-GFP in patient cells (Fig. 2c bottom panel and inset) and performed the laser injury assay using FM4-64 dye. ANO5-GFP expression ameliorated the poor repair phenotype of MMD3 patient myoblasts: their ability to exclude dye entry following focal injury became similar to that of healthy cells (Fig. 2c–e). In contrast to ANO5, expressing dysferlin, which rescues the repair deficit in LGMD2B cells, did not reverse the PMR defect in patient myoblasts (Supplemental Fig. 2a). These results with two different patient cell lineages (myoblasts and fibroblasts) and rescue by re-expression of ANO5 establish the requirement of ANO5 for cell membrane repair in MMD3 patients. The results also indicate that unlike GFP fusions that affect the activity of other TMEM16 proteins^15^, the ANO5-GFP fusion protein is functional. Further, as can be expected^33,34^ ANO5 plays a role distinct from that of dysferlin in membrane repair.

ANO5 has been localized to the ER in a variety of non-muscle cells^18^. As ANO5-GFP is functional in terms of its ability to rescue the membrane-repair phenotype of patient myoblasts (Fig. 2c–e), we used it to investigate ANO5 localization in a human cell line (HER-911). ANO5-GFP localized to the ER in these cells (Supplemental Fig. 2b). Similarly, co-expression of ANO5-GFP with an RFP-tagged ER luminal marker (RFP-KDEL or ER-RFP) in healthy human myoblasts showed overlapping reticular staining of both proteins, confirming ER localization of ANO5 in human myoblasts (Fig. 2f). Fluorescence recovery after photobleaching measurements of ER membrane-localized membrane proteins Sec61-mCherry, ANO5-GFP showed similar recovery kinetics indicating that ANO5-GFP localizes to the ER membrane and is not trapped in the ER due to misfolding or other potential artefacts (Fig. 2g, h).

### ANO5 accumulates at site of repair and helps maintain ER structural integrity

**Fig. 3:**
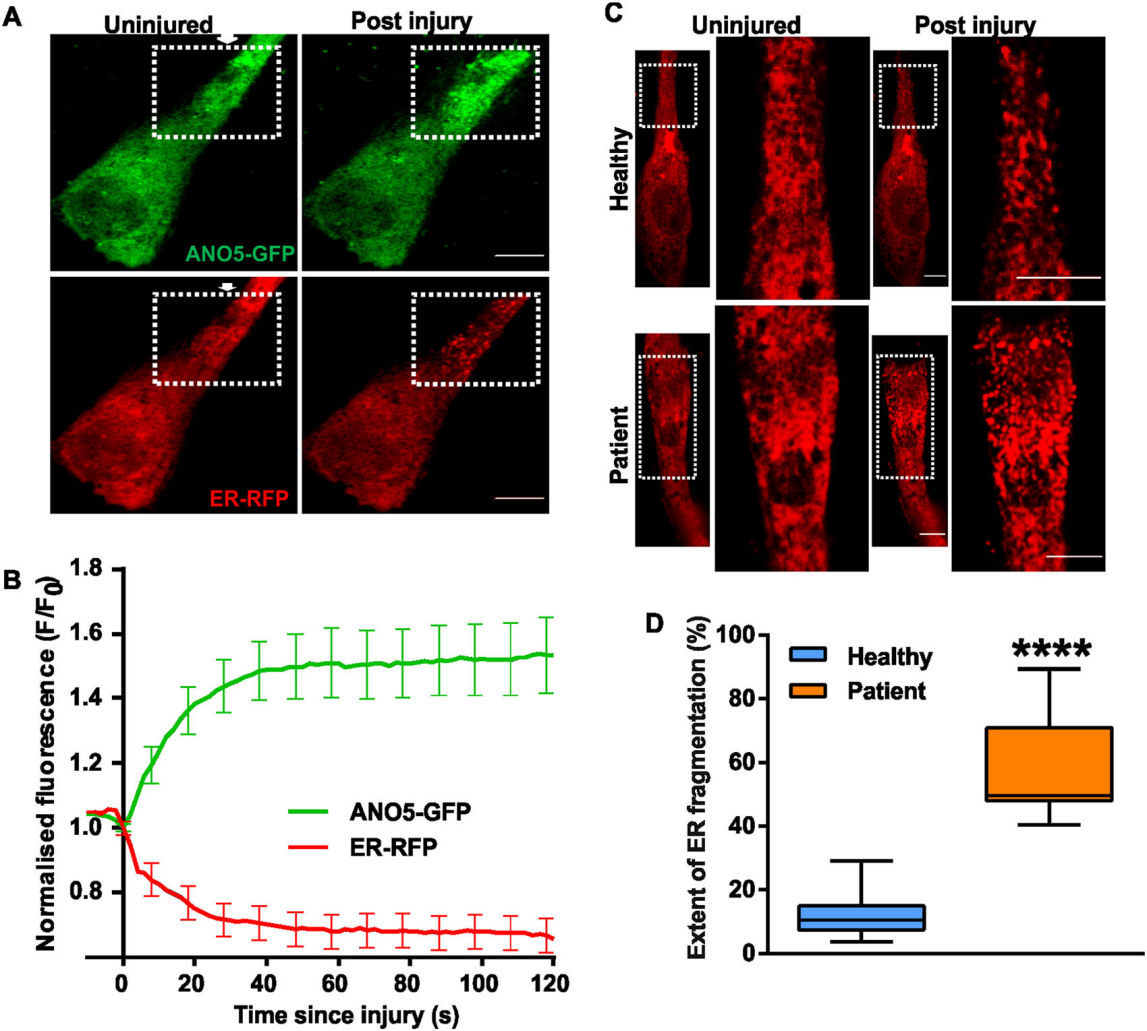
Effect of injury on the ANO5 distribution and ER structure. **a** Images of healthy myoblasts expressing ANO5-GFP and RFP-KDEL are injured (at site marked by the arrow) by laser beam. Representative images before and 60 s after the injury are shown. **b** Kinetics of ANO5-GFP accumulation and RFP-KDEL loss at the site of repair (eg; region marked in panel **a**) is quantified in the plot. Scale bar = 10 µm. *n* = 18 cells **c** ANO5 patient myoblasts expressing RFP-KDEL showed significantly greater ER fragmentation (marked by the dotted box, and presented in the zoomed image). **d** Quantification of ER fragmentation in healthy and patient myoblasts showed that lack of ANO5 causes increased ER fragmentation following injury; *n* = 13 cells (healthy) and 9 cells (Patient) (****p < 0.0001). Scale bar = 10 µm

To study the involvement of ANO5 in PMR, we imaged ANO5-GFP and the luminal ER marker RFP-KDEL in healthy human myoblasts following focal laser injury. Interestingly, ANO5-GFP selectively accumulated at the site of injury, even as the ER at the injury site appeared to fragment, as indicated by the luminal marker RFP-KDEL (Fig. 3a, b). The injury-induced loss in ER structural integrity was associated with leakage of the ER luminal marker RFP-KDEL, indicated by the decrease in RFP signal (Fig. 3b). Acute ER fragmentation is triggered by persistent increases in cytosolic calcium ([Ca^2+^]_c_), generated by exposing cells to purinergic signals or to the Ca^2+^ ionophore ionomycin, in the presence of extracellular Ca^2+^ ^38^. ER localized Ca^2+^-activated chloride channels (CaCCs) are required for regulating cytosolic calcium in response to purinergic signaling in epithelial cells^39^. Considering the ER localization and Ca^2+^-activated non-selective ion channel activity of ANO5^22,25,26^, we hypothesized that ANO5 may mitigate the rise in ([Ca^2+^]_c_ resulting from focal injury, thus maintaining ER integrity in injured cells. Using RFP-KDEL we observed that while focal injury of healthy cells caused about 10% of the cellular ER near the injury site was fragmented. Use of ER membrane marker Sec61-mCherry independently confirmed fragmentation of the ER near the injury site (Supplemental video 3). However, in MMD3 patient myoblasts focal injury resulted in fragmentation of nearly half of all of the cellular ER (Fig. 3c, d, Supplemental videos 1, 2). Furthermore, total internal reflection fluorescence (TIRF) microscopy to monitor plasma membrane proximal ER^40^ revealed a ~2-fold reduction in RFP-KDEL signal in the vicinity of the PM in uninjured patient myoblasts compared with healthy myoblasts (Supplemental Fig. 3). Taken together, these findings suggest a role of ANO5 in maintaining the plasma membrane proximity of ER in resting cells and integrity of the ER in the injured cells, possibly by playing a role in [Ca^2+^]_c_ homeostasis through its ion channel activity.

### Modeling 3-dimensional structure of ANO5 protein

**Fig. 4:**
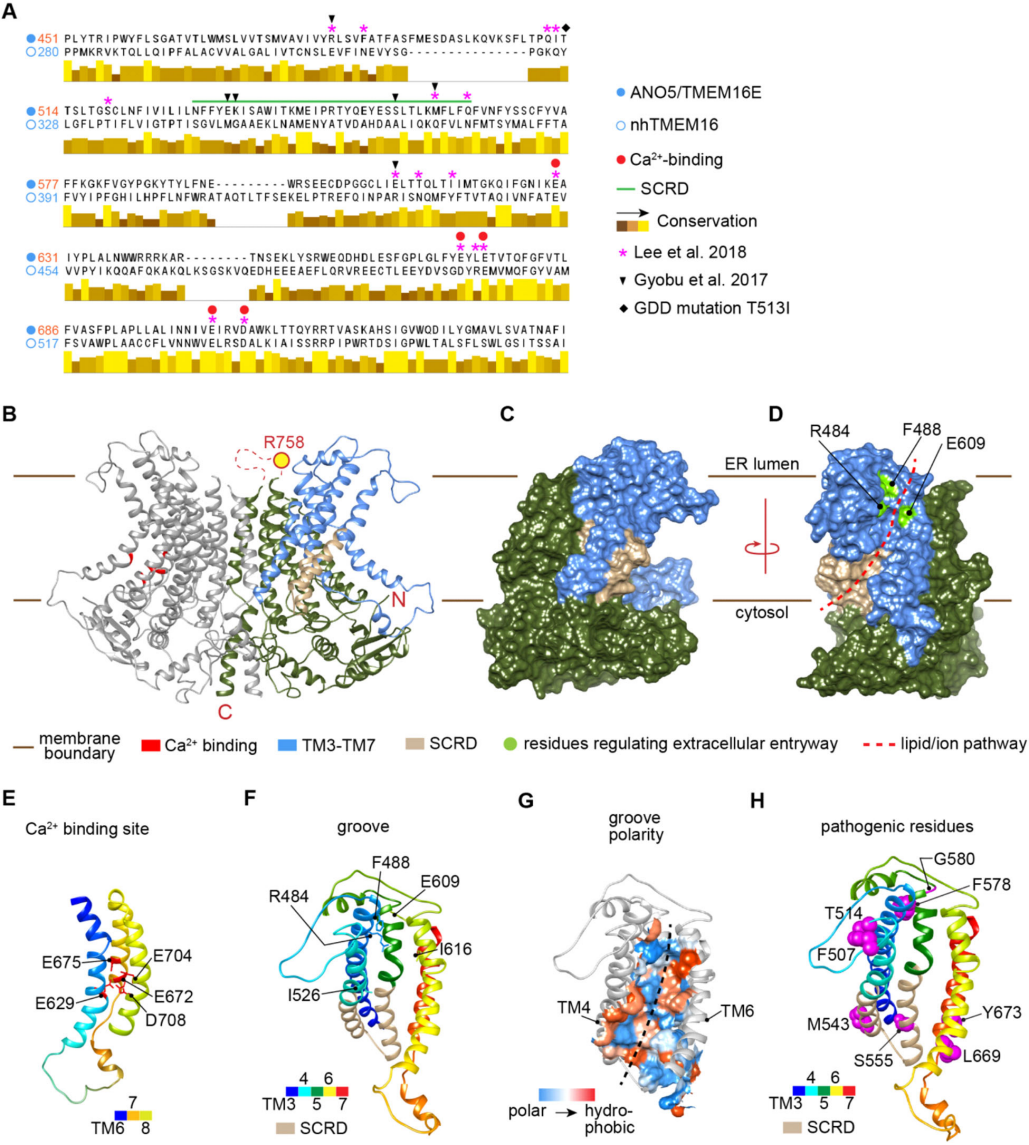
Structural model of ANO5 based on nhTMEM structure. **a** Partial sequence alignment of human ANO5 and nhTMEM16. The scramblase domain (SCRD, corresponding to N530….Q564 of ANO5) is indicated with a green bar. Asterisks indicate residues in nhTMEM16 identified as being important for function^14^ whereas arrowheads indicate residues that were found to be important for the scramblase activity of mouse ANO5/TMEM16E^21^. Red filled circles show residues implicated in Ca^2+^ binding (see also **b** and **e**) and the black diamond indicates a residue implicated in gnathodiaphyseal dysplasia (GDD). **b** Homology model of ANO5 based on nhTMEM16 (PDB ID: 4WIS^12^) is shown in ribbon representation. The protein is shown as a homodimer in the plane of the ER membrane (the lumenal and cytosolic faces are indicated). Large loops on the extracellular side (notably the loop connecting TM9 and TM10) have been omitted for clarity; also omitted is the C-terminal portion on the cytoplasmic side of the protein. One monomer is shaded in light grey; the other monomer is colored in olive green with TM helices 3-7 highlighted in cornflower blue to show the portion of the protein involved in creating the transmembrane groove or subunit cavity that is implicated in ion and lipid transport across the membrane. Residues that make up the Ca^2+^-binding site are shown in red the monomer at left; the scramblase domain (SCRD) is shown in tan in the right monomer. For clarity, different regions are highlighted in different monomers. **c** Surface representation of the right monomer using the same color coding as in the ribbon representation. **d**–**h** View of the protein after rotation by 90° in the plane of the membrane. **d** Surface representation revealing the transmembrane groove and permeation pathway (dashed line). Residues that regulate lipid and ion entry on the extracellular side are indicated in chartreuse green (see also **f**). **e** Acidic residues located on TM6, 7 and 8 that make up the Ca^2+^-binding site are indicated. For **e**, **f**, and **g**, the ribbon is color coded from N-terminal to C-terminal from blue to red via green and yellow. **f** View of the ion/lipid transporting groove. TM helices 3-7 (blue in **b**) are shown with the SCRD domain (cytoplasmic side of TM4-TM5) colored tan; residues important for regulating access to the transport pathway from the extracellular side are indicated (see also **d**). **g** Surface representation of the transmembrane groove of ANO5, color coded to reveal polarity. **h** View of the ANO5 groove illustrating the location of residues known to be associated with pathological phenotypes

The results above suggest that ANO5 may play a role in cellular Ca^2+^ homeostasis by functioning as a Ca^2+^-dependent ion channel in the ER. As a preliminary test of this idea we asked whether patient mutations map onto regions of ANO5 that are predicted to be involved in its transport function. As ANO5 has not been structurally characterized we first built a homology model of the protein templated on the structure of *Nectria haematococca* TMEM16 (nhTMEM16), an ancestral structurally defined ion channel and phospholipid scramblase of the TMEM16 protein family. We carried out a structure-based alignment of the primary sequence of ANO5 with nhTMEM16 using HHPred (Supplemental Fig. 4), and then used MODELLER to build a 3D model. The alignment located the previously identified SCRD domain as well as several other residues that are known to be important for phospholipid scrambling and ion transport activities in ANO5 and nhTMEM16 (Fig. 4a)^14,21^. The 3D structural model, placed in the context of the ER membrane consistent with the known localization of the protein (Fig. 4b–d), depicts ANO5 as a homodimer in which each monomer has 10 transmembrane (TM) segments (Fig. 4b). TM10 contributes significantly to the dimer interface. The Ca^2+^ binding site in each monomer is buried within the protein but accessible from the cytoplasmic side (shown only in the monomer on the left, Fig. 4b, and in more detail in Fig. 4e). The cytoplasmic C-terminal portion of the protein, as well as unstructured loops, especially a large loop between TM9 and TM10 (in the ER lumen), are not shown for simplicity. Rotation of the ANO5 dimer by 90° in the plane of the membrane reveals the membrane-facing, hydrophilic transmembrane groove (subunit cavity) within each monomer that is implicated in lipid and ion transport (Fig. 4c, d; the suggested permeation pathway is shown by the dashed line in Fig. 4d). The groove is formed from elements of TM helices 3–7; it contains the SCRD on its cytoplasmic side (Fig. 4c, d, f), as well as a number of functionally important residues that control access to the transmembrane pathway from the ER lumen. These residues are noted in the sequence alignment (Fig. 4a) and their location in the 3D model of ANO5 protein is shown in Fig. 4f. The groove has a significantly polar surface consistent with its function in lipid and ion transport (Fig. 4g) but is somewhat wider in ANO5 than in nhTMEM16, consistent with the ability of ANO5 to transport the large cation N-methyl-D-Glucamine (NMDG)^26^. Of interest, TM3 and TM6 are tethered by a pair of charged residues in nhTMEM16 (E313 (TM3) and R432 (TM6)) and this feature is replicated in the 3D structural model of ANO5 (R484 (TM3) and E609 (TM6)). Disruption of the interaction between these charged residues has been proposed to be part of a series of conformational changes, including rotation of TM6, that open the lipid/ion transport pathway^14^ Thus, substitution of R484 and E609 with tryptophan is predicted to disrupt transport activity as seen for the corresponding E313W and R432W mutations in nhTMEM16. Many pathogenic mutations in ANO5 are found near the transmembrane groove and Ca^2+^ binding site as shown in (Fig. 4e, h). Thus, M543 and S555 are located within the SCRD, and Y673 is adjacent to the E672 residue implicated in Ca^2+^ binding. The corresponding patient mutations include M543N, S555I, S555R, and Y673C. Residues T513 and T514 are also of interest, the former being implicated in GDD while the latter is associated with adult onset muscular dystrophy. Both are at the ER lumenal end of TM4 (Fig. 4a, h).

### ANO5 is required for buffering the increase in cytosolic Ca^2+^

**Fig. 5:**
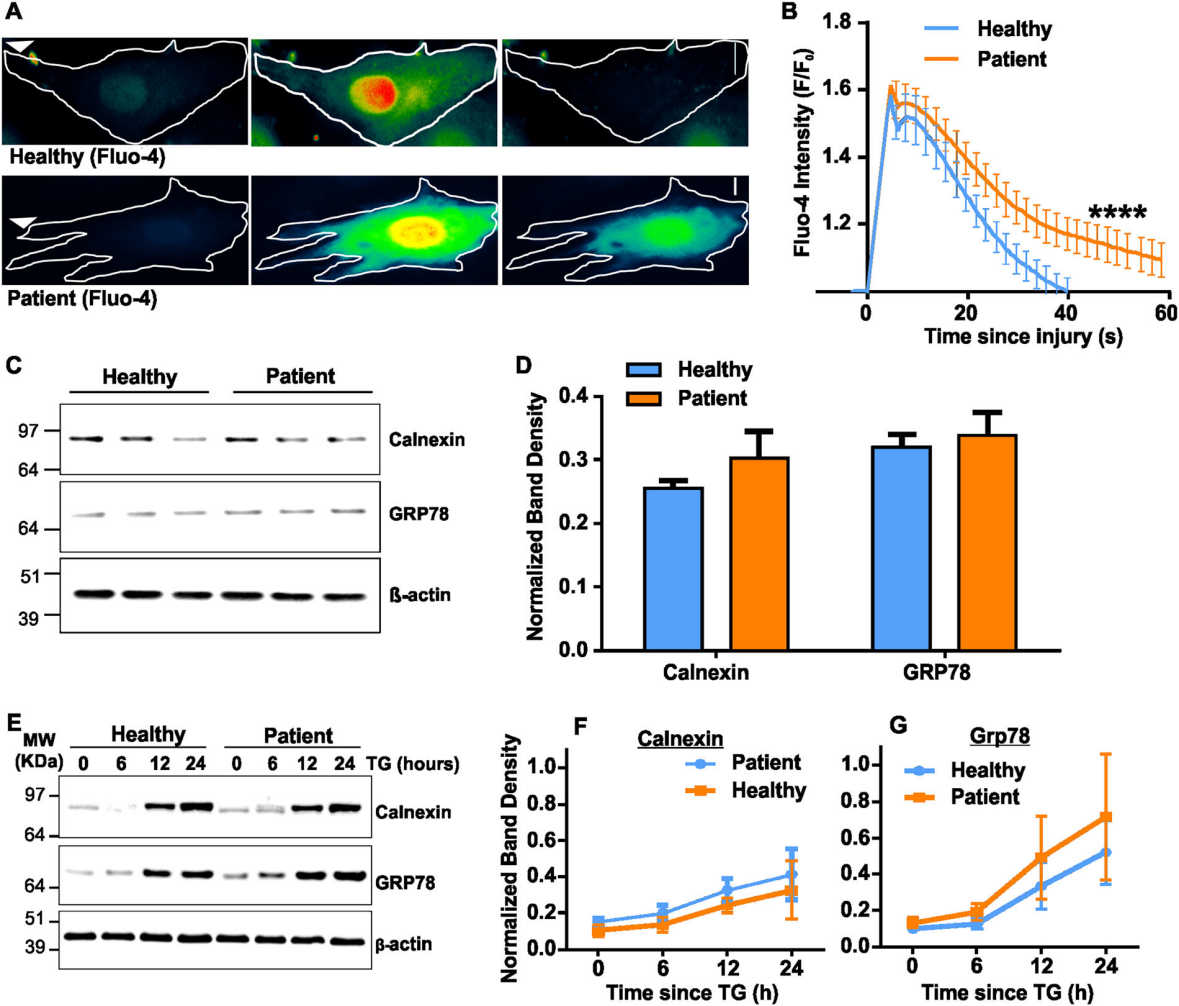
ANO5 deficit causes [Ca^2+^]_c_ overload in injured cells without affecting ER stress response. **a** Psuedocolor images showing measurement of [Ca^2+^]_c_ by Fluo-4 following PM injury in healthy and patient myoblasts. **b** Time course of Fluo-4 fluorescence (ΔF/F0) in healthy and Ano5 myoblasts. Ano5 patient myoblasts took significantly longer time to clear the [Ca^2+^]_c_ increases following cell injury than that of healthy myoblasts. Fluo4 fluorescence was calculated for different groups as mentioned and presented as mean ± SEM. *N* = 36 cells (healthy) and 46 cells (patient) (****p < 0.0001). **c**–**g** Analyses of ER stress response in the human myoblasts in basal and induced states. **c** Western blot image and **d** densitometric quantification of normalized band intensities (*n* = 3 independent blots) in untreated healthy and ANO5 patient myoblasts. **e** Western blot image and **f**, **g** kinetic plots of normalized band intensities (*n* = 3 independent blots; mean ± SEM) for **f** calnexin and **g** GRP78 following induction of ER stress for the indicated time periods by treatment with ER calcium modulator thapsigargin (TG)

The ER-localized Ca^2+^-activated ion channel bestrophin regulates increases in [Ca^2+^]_c_ caused by purinergic and other signaling pathways in epithelial cells^39^. We therefore examined if greater injury-triggered ER fragmentation in ANO5-deficient patient myoblasts could be due to a poor handling of elevated [Ca^2+^]_c_ by the patient myoblasts. We used the Ca^2+^-sensitive dye Fluo-4 to measure cytosolic Ca^2+^ change^41^ induced either by a purinergic signal (ATP) or injury. ATP induced a ~20% increase in [Ca^2+^]_c_ in healthy cells, which returned to baseline over 15 min. However, in patient cells ATP induced a more sustained [Ca^2+^]_c_ increase, followed by a slower return to baseline, resulting in [Ca^2+^]_c_ overload (Supplemental Fig. 5a–d). Similar to the effect seen with ATP, injury-triggered increase of [Ca^2+^]_c_ in the patient cells was also cleared slowly (Fig. 5a, b). While healthy myoblasts attained peak [Ca^2+^]_c_ levels within 10 s following injury, and cleared this within 40 s (Fig. 5a, upper panel and b, blue trace), ANO5 deficient patient myoblasts took longer (>60 s) to clear the [Ca^2+^]_c_ elevation (Fig. 5a, lower panel and b, red trace). Thus, dysregulation of Ca^2+^ homeostasis in patient myoblasts, specifically in the time to return to baseline through clearance of the [Ca^2+^]_c_ following signal- or injury-mediated elevation, correlates with the increased ER fragmentation in the injured patient cells (Fig. 3c, d).

ER Ca^2+^ dysregulation causes ER stress, leading to muscle pathology^42,43^. However, we found that calnexin and GRP78 (molecular chaperones upregulated by ER stress) levels are similar in healthy and patient myoblasts (Fig. 5c, d) and treatment of myoblasts with the ER stress inducer thapsigargin (TG; 1 mM TG for 0–24 h) caused a similar increase in calnexin and GRP78 levels in healthy and patient myoblasts (Fig. 5e–g). Thus, lack of ANO5 neither enhances ER stress nor alters the ability of the patient muscle cells to respond to ER stress.

### Efficient buffering of injury-triggered cytosolic Ca^2+^ increase improves patient myoblast repair

**Fig. 6:**
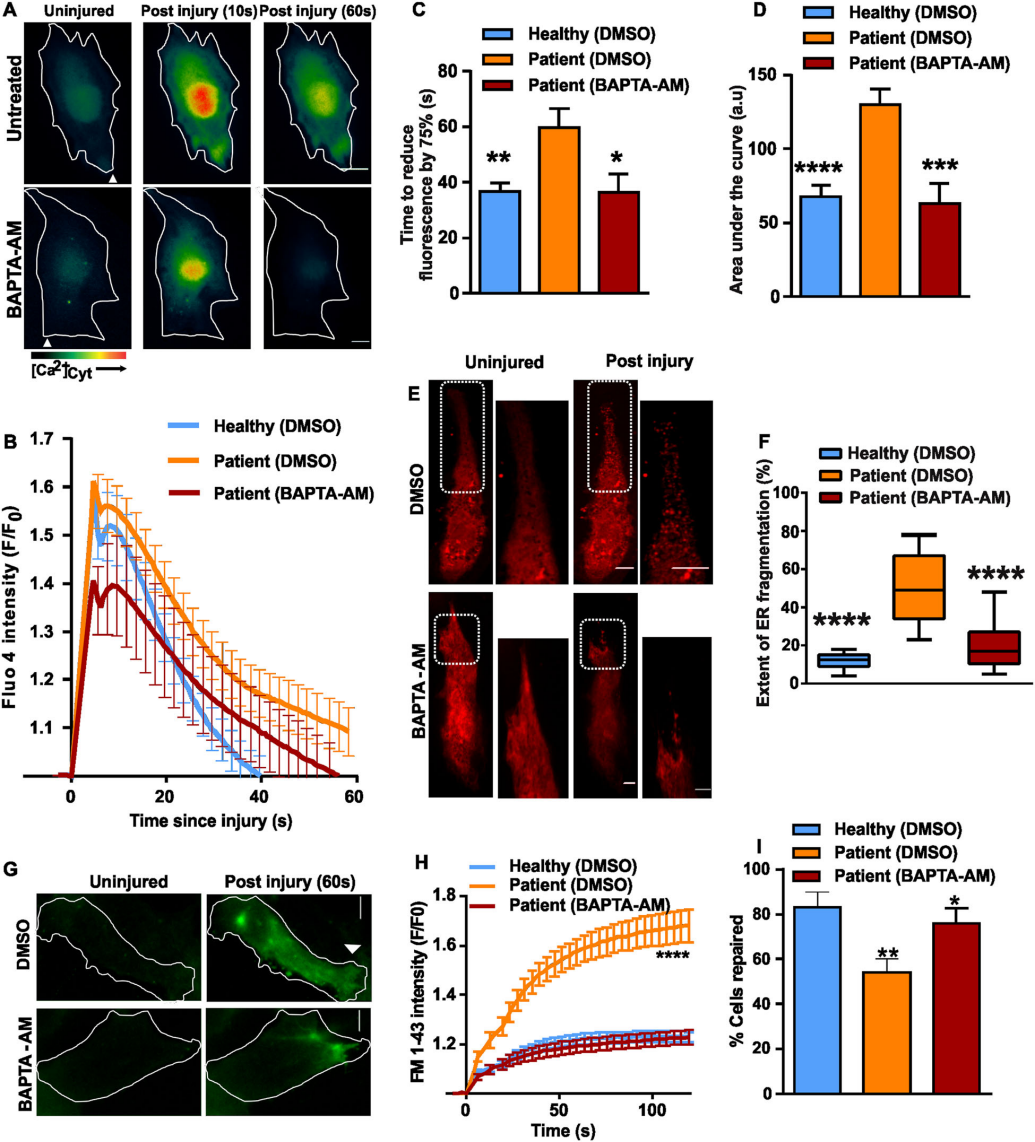
Preventing cytosolic calcium overload ameliorates PMR defect in ANO5 deficient cells. **a** Cytoplasmic Ca^2+^ chelator, BAPTA-AM (10 µM) was applied to Fluo-4 loaded patient myoblasts. The pseudo-colored images show the change in Fluo-4 fluorescence following plasma membrane injury. **b** Kinetics of change in Fluo-4 fluorescence in BAPTA-AM/DMSO-treated patient and healthy myoblasts. *n* = 16 (Healthy - DMSO), 19 (Patient - DMSO), 13 (Patient - BAPTA-AM), **c** Time for the peak Fluo-4 fluorescence to reduce by 75% (*p = 0.0194, **p = 0.0035) and **d** Area under the Fluo-4 kinetics plot was calculated for the various groups of cells and expressed as mean ± SEM. ***p = 0.0006, ****p < 0.0001. **e** Images and **f** quantification of extent of ER fragmentation assessed in patient myoblasts expressing RFP-KDEL and preincubated with DMSO/BAPTA-AM. In **e**, the boxed region is shown in the zoomed images. *n* = 26 cells (Healthy - DMSO), 31 cells (Patient - DMSO) and 41 cells (Patient - BAPTA-AM) from two independent experiments (****p < 0.0001). **g** Images showing FM1-43 dye entry following PM injury in patient myoblasts treated with DMSO or BAPTA-AM. **h** Kinetics of the FM1-43 dye entry in patient and healthy myoblasts treated as indicated. *n* = 15 (Healthy - DMSO), 19 (Patient - DMSO), 34 (Patient - BAPTA-AM) (****p < 0.0001). **I** Quantification of the fraction of injured cells that repaired based on the FM1-43 dye entry assay, **p = 0.0065, *p = 0.0237. The data is from two independent experiments

Above results indicate that ANO5 deficiency dysregulates the clearance of injury-triggered elevation in [Ca^2+^]_c_, leading to increased ER fragmentation in injured cells. We thus hypothesized that it should be possible to correct this deficit by artificially buffering [Ca^2+^]_c_. To test this idea we loaded patient muscle cells with the cell permeant calcium chelator BAPTA-AM. The presence of BAPTA in the patient cell cytosol, corrected the rate at which the injury-triggered increase in [Ca^2+^]_c_ in the patient myoblasts returned to baseline (Fig. 6a–c) and also reduced the total [Ca^2+^]_c_ load (area under the curve for injury-triggered increase in Fluo-4 fluorescence) (Fig. 6b, d). We then examined ER fragmentation using RFP-KDEL in BAPTA-AM-treated patient myoblasts, which showed 2.5-fold lower ER fragmentation at the site of injury compared to vehicle-treated cells, making this comparable to the extent of ER fragmentation in healthy myoblasts (Fig. 6e, f).

Next, we tested if BAPTA-AM-treatment affects PMR kinetics. We found that BAPTA-AM treatment reversed the excessive FM1-43 dye entry following focal laser injury of plasma membrane of patient myoblasts (Fig. 6g, h). This reversal of the excessive dye entry in injured patient myoblasts enabled patient cells to repair in numbers that are comparable to the healthy human myoblasts (Fig. 6i). These data link several features of ANO5 deficiency in myoblasts. Upon focal injury, the patient cells display poor PMR, increased ER fragmentation and dysregulated Ca^2+^ homeostasis, which we hypothesize results in the poor PMR.

## Discussion

We present the first human muscle cell model to examine the cellular function of ANO5 in muscular dystrophy. We generated myoblasts from a MMD3 patient with the c.2272C>T mutation in the *ANO5* gene. This mutation corresponds to an R758C substitution in the protein, causing it to be expressed at significantly reduced levels. Our 3D structural model of ANO5 places R758 in an ER-luminal loop between TM9 and TM10 (Fig. 4b) where it may play a role in establishing the dimerization interface. The R758C substitution may destabilize the protein, accounting for its low steady state level. Further work will be needed to clarify this point.

Using patient-derived myoblasts we find that while ANO5 is required for PMR, it is not required for myoblast fusion and myogenesis as has been reported for an ANO5-deficient mouse model^26,28^. While this difference between the mouse and human cells could reflect a species-specific myogenic role of ANO5, unrelated ANO5 knockout mouse models also show no developmental or regenerative myogenic deficit^19,35^. Furthermore, patients with ANO5 mutations do not exhibit developmental myogenic deficit. Thus, our results do not support a myogenic role of ANO5 in humans, but, just as in mouse muscles^28^, they demonstrate a requirement for ANO5 in human muscle cell sarcolemmal repair.

Unlike the sarcolemmal repair protein dysferlin, ANO5 plays a non-redundant role in sarcolemmal repair (Fig. 2). ANO5 expression fails to reverse the PMR defect in dysferlin deficient muscle fibers^34^ and we have shown that dysferlin enables PMR by facilitating lysosome docking and fusion^33^. ANO5-deficient patient fibroblasts have normal injury-triggered lysosome exocytosis^29^ and we find ANO5 deficit increases ER fragmentation following injury (Fig. 3). This implicates ANO5 in regulation of ER physiology. A TMEM16 protein family member Ist2 regulates ER morphology and ER-plasma membrane association in yeast^44^ while another member - ANO8 facilitates this in mammalian cells^45^. ER-PM tethering is crucial for cellular calcium homeostasis in mammalian cells^46^. As ER fragmentation is linked to persistent increases in [Ca^2+^]_c_, this observation further suggests a role for ANO5 in ER-mediated [Ca^2+^]_c_ homeostasis. Consistent with this we find that controlling injury-triggered [Ca^2+^]_c_ increase improves PMR in the patient cells. ANO5 is proposed to have both ion channel and phospholipid scramblase activities^19,21,28^, but this remains to be verified by direct assay of purified ANO5 in a reconstituted system. Interestingly, our 3D structural model of ANO5 places a number of disease-linked mutations close to the site at which activating Ca^2+^ ions bind the protein, and also in the vicinity of the SCRD and the lumenal entryway to the ion/lipid transport pathway (Fig. 4a, h). Our studies show that restoring the Ca^2+^ homeostasis is more important for the health of the patient muscle cells, while the relevance of the proposed scramblase function remains unclear.

The ER is the major intracellular Ca^2+^ store and the most likely organelle to normalize elevated [Ca^2+^]_c_ caused by plasma membrane injury. Ca^2+^ uptake across the ER is electrically neutral^47^, it must be accompanied by, for example, the expulsion of K^+^ or H^+^ ions from the ER and/or the import of Cl^−^ into the ER lumen. The SERCA channel, important for excitation-contraction coupling in muscle, is proposed to accomplish electroneutrality by exchanging Ca^2+^ for H^+^^48^. However, it is unclear whether SERCA alone can handle the surge in [Ca^2+^]_c_ caused by injury and our data point to an important role for ANO5 in this process. Thus, the proposed channel activity of ANO5^22,26^ could allow rapid electroneutral Ca^2+^ exchange through the counter-ion Cl^−^. This is consistent with the reported deficit in ER Ca^2+^ influx due to poor Cl^−^ transport across the ER membrane^47^.

In Summary, our data suggest that ANO5 influences cytosolic Ca^2+^ homeostasis by way of its ER-localized ion channel activity. This mode of action of ANO5 reveals a new membrane repair mechanism that depends on coping with the cytosolic Ca^2+^ increase that accompanies membrane injury. While other details of this process are subject of future studies, our finding that buffering of excess cytosolic Ca^2+^ improves repair of ANO5-patient muscle cells provides proof-of-principle for efficient removal of cytosolic Ca^2+^ in the muscle of ANO5 patients as a potential therapeutic strategy for treating LGMD2L and MMD3 patients.

## Methods

### Human myoblasts

Myoblast cultures were generated from muscle biopsy obtained from following ethical approval by Helsinki university human subject ethics committee and informed consent by the donor. The patient cells were obtained from the quadriceps of a 46-year-old Caucasian male with confirmed homozygous C.2272>T mutation in the *ANO5* gene. The patient had disease symptoms since prior to the age of 25 years old, which includes elevated serum CK levels (12,000–16,000 U/L). The healthy cells were obtained from semi-tendonitis of a 25 year old Caucasian male donor with no known muscle disease or damage. All human samples were obtained with informed consent from each participant. Primary myoblasts were purified by magnetic activated cell sorting using anti-Cluster Designate 56 (CD 56 - a specific marker of myoblasts) coated beads (MACS, Miltenyl Biotech). Immortalization was performed using human telomerase reverse transcriptase (hTERT, to preserve telomere length) and cyclin-dependent kinase-4 (cdk-4, to overcome the p16 pathway) transgenes, as described previously^49^. The cells were cultured in Skeletal Muscle Cell Basal Media (PromoCell GmbH, Heidelberg, Germany) containing 15% fetal bovine serum, 5% fetal calf serum and necessary supplements (hEGF, hbFGF, fetuin, insulin and dexamethasone). Hundred percent confluent myoblasts cultures were differentiated using Skeletal Muscle Cell Differentiation Medium (PromoCell GmbH, Heidelberg, Germany) with the differentiation supplements, as described previously^50^. Transfections were performed in OPTI-MEM (Invitrogen) using Lipofectamine LTX for 4 h followed by addition of full growth media and growing the cells for 24–48 h. LGMD2L fibroblasts were used, as described previously^29^. All the cell culture experiments were repeated using at least three independent cell cultures or as indicated in the figure legends.

### Injury assays

These assays were performed as described previously^33,37^.

#### Glass bead injury

Cells cultured on coverslips were transferred to cell imaging media (CIM) or phosphate buffered saline (PBS) (Sigma-Aldrich) containing 2 mg/ml of lysine-fixable FITC-dextran (Life Technologies). Cells were injured by rolling glass beads (Sigma-Aldrich) over the cells. They were allowed to heal at 37 1C for 5 min, and then incubated at 37 °C for 5 min in CIM/PBS buffer containing 2 mg/ml of lysine-fixable TRITC dextran (Life Technologies). Cells were fixed in 4% PFA, and nuclei were counterstained with Hoechst 33342; the cells were then mounted in fluorescence mounting medium (Dako) and imaged as described in Supplemental Methods. The number of FITC-positive cells (injured and repaired) and TRITC-positive cells (injured and not repaired) were counted. The number of injured cells that failed to be repaired was expressed as a percentage of the total injured cells.

#### Laser injury

Cells cultured on coverslips were transferred to and incubated in CIM/PBS buffer with 1 mg/ml FM1-43 dye (Life Technologies) and placed in a Tokai Hit microscopy stage-top ZILCS incubator (Tokai Hit Co., Fujinomiya-shi, Japan) maintained at 37 °C. For laser injury, a 1- to 5-µm^2^ area was irradiated for 10 ms with a pulsed laser (Ablate!, 3i Intelligent Imaging Innovations, Inc. Denver, CO, USA). Cells were imaged at 2 s intervals using IX81 Olympus microscope (Olympus America, Center Valley, PA, USA). FM dye intensity (ΔF/F where F is the original value) was used to quantify the kinetics of PMR and represented with intervals of five frames. For monitoring ER fragmentation, cells expressing ER-RFP or Sec61-mCherry were imaged live by spinning disc confocal microscopy as the plasma membrane was injured focally by pulsed laser. The longest axis of the cell was drawn from the point of injury to the opposite end of the cell indicating the total length of the cell (Supplemental Fig. 6) and the length of portion of the cell where the tubular appearance of the ER was lost following injury and resulted in the punctate (bead-on-string) appearance was measured as the fragmented length of the cell (Supplemental Fig. 6). The percentage of the total length of the cell where injury caused the ER fragmentation was quantified for multiple cells and presented as mean (±SEM).

### Fluorescence recovery after photobleaching

Immortalized myoblasts were grown on coverslips 24–48 h prior to imaging at upwards of 50% confluency at the time of imaging. Coverslips were mounted on holders and washed in Dulbecco’s PBS (DPBS) and cells were imaged live using a 488-nm (for GFP) or 561-nm (for mCherry) lasers attached to the Hamamatsu spinning sisc confocal microscope on an Olympus IX81 stand. Cells were photobleached using the imaging laser and fluorescence recovery was monitored over time. Results were analyzed using Slidebook 6.0. The data for each condition were recorded, and recovery kinetics were analyzed and plotted.

### Immunocytochemistry

Cells were fixed in 4% PFA and blocked with 2% BSA and 5% Goat serum in PBS for an hour at room temperature. Primary antibody incubations were followed by 1 h incubation with Alexa 488 or 594 goat secondary antibodies (Invitrogen, 1/1000) from the adequate species. Coverslips were mounted in Fluoromount G (Southern Biotech, Birmingham, USA) and examined through a Leica confocal microscope (TCS SP2.AOBS microscope, Leica, Germany) using the 488 nm and the 594 nm lines of an argon laser. The antibodies used for immuno-staining were Anti-ANO5 (NIH/UC Davis), Anti-MYH3 (Santacruz Biotechnology Inc).

### Western Blotting

Cells were lysed with RIPA buffer (Sigma-Aldrich) containing protease inhibitor cocktail (Fisher Scientific, Waltham, MA, USA). Proteins transferred to nitrocellulose membranes were probed with the indicated antibodies against: Calnexin (Millipore), GRP78 (Santa Cruz) and β-actin (Santa Cruz). Primary antibodies were followed by the appropriate HRP-conjugated secondary antibodies (Sigma-Aldrich), and chemiluminescent western blotting substrate (Fisher, Waltham, MA, USA; GE Healthcare, Pittsburgh, PA, USA). The blots were then processed on Bio-Rad ChemiDoc Touch Imaging System (Bio-Rad).

### Quantitative RT PCR

Real-time qRT-PCR was performed using total RNA (1 µg) from subjected to DNAse I digestion (1 U) in 1 × DNAse I reaction buffer (Promega) by incubating at 37 °C for 30 min to remove genomic DNA contamination. The reaction was inactivated by adding 1 µl of stop solution (Promega) and heating for 10 min at 65 °C. Subsequently, the RNA sample was reverse transcribed to cDNA using Superscript II (Life Technologies) and oligo dT primers. The cDNA thus generated was amplified in triplicates in SYBR Green PCR Master Mix (Life Technologies) using 1 µM of forward and reverse primers specific to each gene and 1 µl of cDNA template in a total volume of 50 µl. Predesigned SYBR Green primer pairs specific to exon 12-13 (Primer 1) and exons 16–17 (Primer 2) of human Anoctamin 5 gene (RefSeqID - NM_01142649) were obtained from Sigma Aldrich. Glyceraldehyde-3-phosphate dehydrogenase (*Gapdh*) was used as internal control and the primers used were (forward) 5′-*CCAGGAGCGAGACCCCACTAACA*-3′ and (reverse) 5′-*TCAAGTGGGCCCCGGCCTT*-3′. The results were independently validated for both primers using 18srRNA as an independent loading control.

### Ca^2+^ measurement assays

For measuring cytosolic Ca^2+^, myoblasts and fibroblasts cultured on coverslips were incubated with DMEM without FBS containing 10 μM of Fluo-4-AM (Life technologies, USA) for 20 min at 37 °C and 5% CO_2_. After washing with prewarmed CIM, cells were submitted to laser injury as described before (ref). To monitor Ca^2+^ influx in the cell following the laser injury, cells were imaged following injury at an interval of 4-6 frames/s for the next 3 min. For every image, the kinetic of Ca^2+^ influx was expressed by the amount of Fluo-4-AM that entered the cell by measuring the cellular Fluo-4-AM emission. For measuring the ER Ca^2+^, we used FRET based biosensor, T1ER^51^. Myoblasts were transfected with T1ER DNA 24 h before assay. Ca^2+^ influx into the cells was induced by PM injury in the presence of 2 mM Ca^2+^.

### Total internal reflection fluorescence (TIRF) microscopy

TIRF was carried out on Olympus IX81 microscope equipped with Lambda DG-4 (Sutter Instruments, Novato CA) widefield illumination system and Evolve 512 EMCCD (Photometrics, Tucson, AZ) camera, with Cell-TIRFTM (Olympus) illuminator and ×60/1.45NA oil objective using Slidebook 6.0.15 (Intelligent Imaging Innovations, Inc. Denver, CO). The cells being imaged were maintained at 37 °C in the Tokai Hit microscope stage top ZILCS incubator (Tokai Hit Co., Japan). For TIRF imaging laser angle was set to obtain penetration depth of 70–120 nm.

### Statistical analysis

For membrane repair analysis the data are presented as averaged values for all of the myoblasts or myofibers used for that analysis. These averaged values were compared with each other using unpaired Student’s t-test. For the densitometric analyses of the western blots, we performed unpaired t-test. For all the other experiments, when the data were not normally distributed or failed the equal variance test, the Mann-Whitney rank sum test in GraphPad Prism 6.0 (GraphPad Software, La Jolla, CA) was performed. Results of all the statistical significance testing for differences are indicated by the asterix symbol, while lack statistical significance is indicated by the absence of this symbol.

### Modeling ANO5 structure

The protein sequence of human ANO5 (Swiss prot ID: Q75V66) was used to search the protein data bank using the HHPred server (https://toolkit.tuebingen.mpg.de/#/tools/hhpred)^52^. Three structurally related homologs were obtained: ANO10 (PDB ID 5OC9), ANO1 (PDB ID 5OYB) and nhTMEM16 (PDB ID 4WIS). The three structures are related in fold and have a root square mean deviation (RMSD) of ~1 Å between them. Since ANO5 contains a scramblase domain (Gyobu (2017) PNAS 114: 6274), further modeling was based on the relatively well characterized nhTMEM16 protein. Thus, the MODELLER server^53^ was used to generate a structural model of ANO5 based on nhTMEM16. The 3D structural model was energy minimized using the GROMOS 43B1 force field embedded in the Swiss PDB viewer tool^54^. The resulting monomer model was then used to generate a dimer model using the nhTMEM16 dimer as a template. The dimer model was energy minimized as above and used to locate residues important for lipid and ion transport and Ca^2+^ binding based on the sequence alignment with nhTMEM16. Selected disease linked mutations identified in ANO5 using the clinvar database^21^ (https://www.ncbi.nlm.nih.gov/clinvar) were mapped onto the structure. Jalview^55^ (http://www.jalview.org) was used to generate the figure showing alignment of ANO5 and nhTMEM16 (Fig. 4a and Supplemental Fig. 4) and USCF chimera^56^ was used to estimate RMSD and generate the figures showing the 3D structural models and residues of importance (Fig. 4b–h).

## Supplementary information

Supplemental Data

Injury-induced ER fragmentation in healthy myoblast

Injury-induced ER fragmentation in an ANO5 patient myoblast

3D reconstruction showing ER following focal injury of a myoblast
