# Supplementary material for: Dysregulated calcium homeostasis prevents plasma membrane repair in Anoctamin 5/TMEM16E-deficient patient muscle cells
Source: Cell Death Discov. 2019 Jul 18;5:118. doi: 10.1038/s41420-019-0197-z (PMC6639303; doi:10.1038/s41420-019-0197-z)
Supplement: Supplementary file 1 — Supplemental Data [file 41420_2019_197_MOESM1_ESM.pdf]

## Supplemental Figures and Legends:

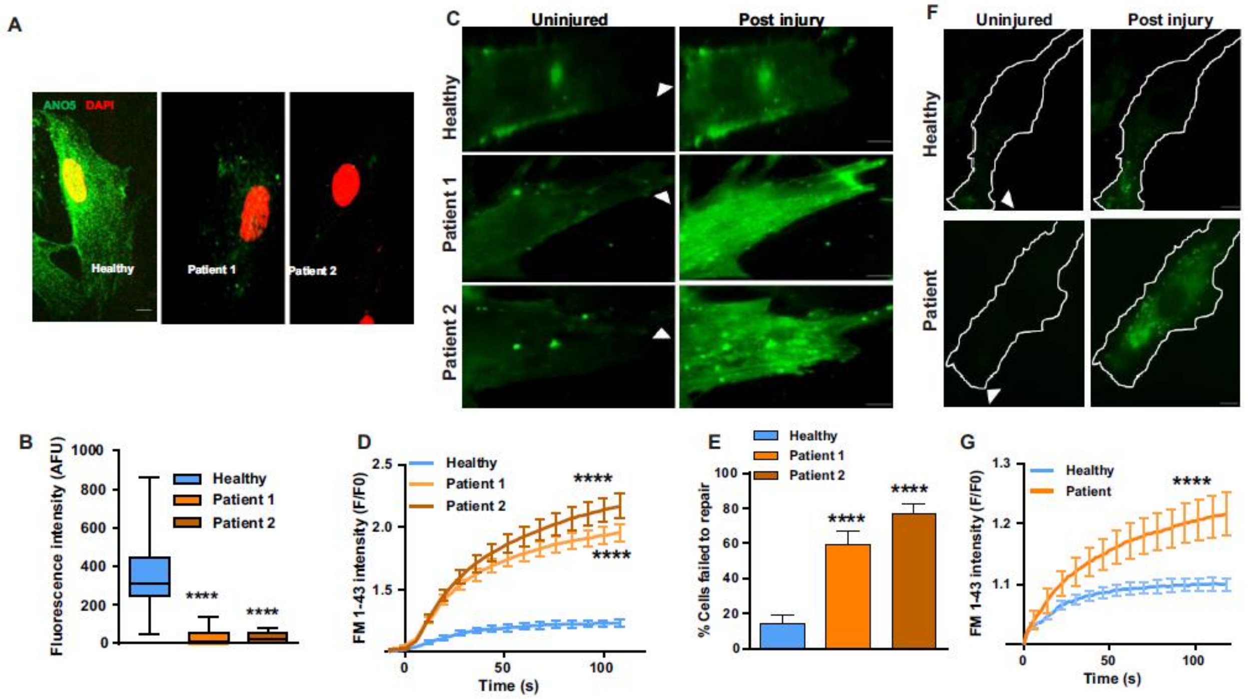


**Supplemental Figure 1: MMD3 patient fibroblast and myoblast lack ANO5 and show poor membrane repair.** (**A**) Images showing fibroblasts isolated from a healthy volunteer or two MMD3 patients stained with Anti-ANO5 antibody. (**B**) Quantification of ANO5 immunostaining in fibroblasts from MMD3 patients. n =13 (healthy), 28 (patient 1) and 17 (patient 2). ****p<0.0001. (**C-E**) FM1-43 dye-based laser injury assay of healthy and patient fibroblasts shows poor plasma membrane repair ability of the patient cells. (**C**) Representative images of individual cells (**D**) Kinetics of FM1-43 dye fluorescence indicating the dye entry kinetics following focal injury. n =59 (healthy), 60 (patient 1) and 40 (patient 2). ****p<0.0001. (**E**) Quantification of the fraction of cells that repaired following laser injury. n ≥ 20 cells in each group from two independent experiments. ****p<0.0001.. (**F**) Images **(G)** Quantification of FM1-43 dye entry into individual healthy

and patient myoblasts during laser injury assay n =45 (healthy), 35 (patient) (****p<0.0001). Scale bar = 10 µm.

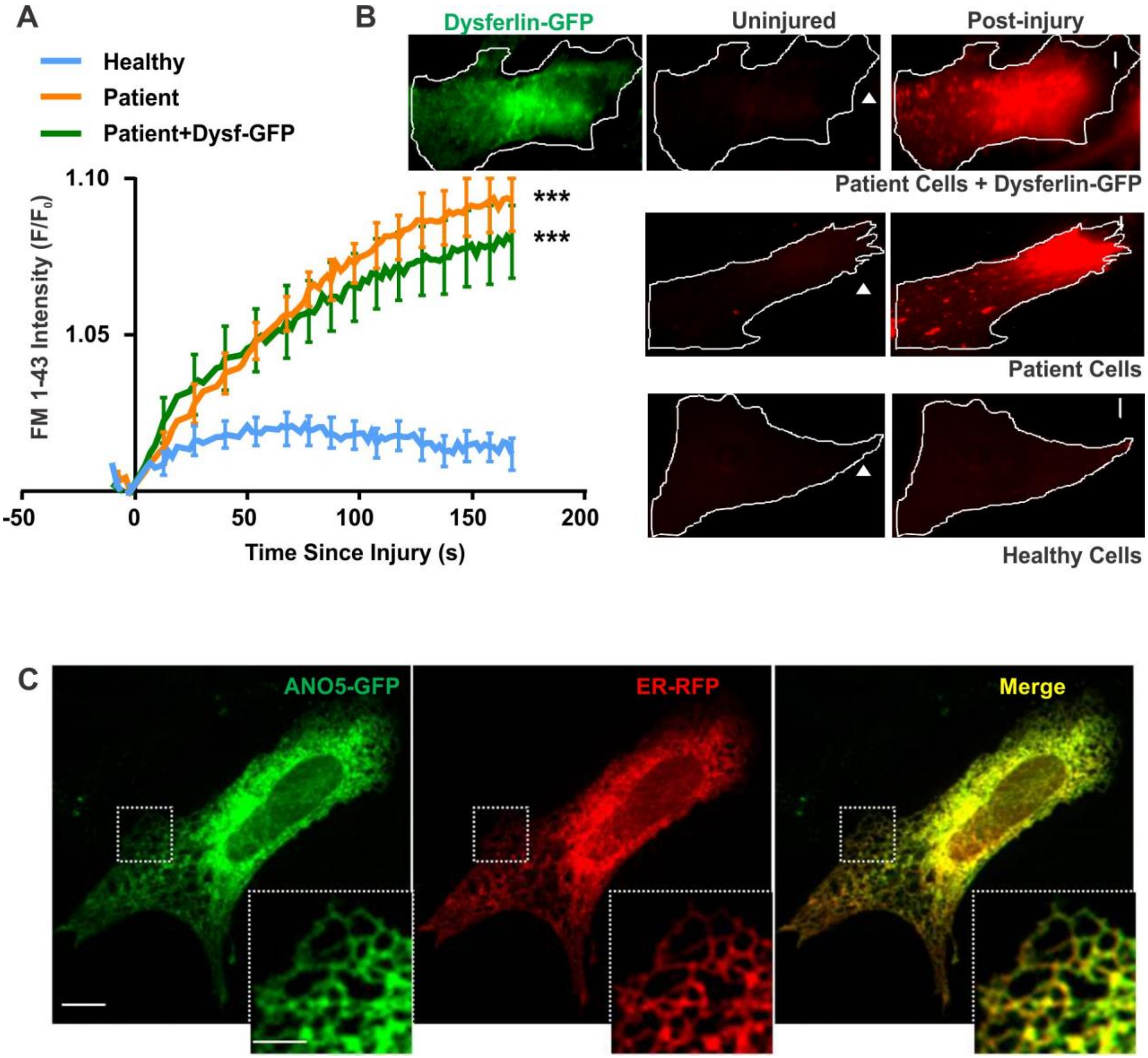


**Supplemental Figure 2: Failure of dysferlin to enable membrane repair in Ano5 patient myoblasts and ER localization of Ano5.** (**A**) Kinetics of laser injury-triggered entry of FM1-43 dye in healthy and Ano5 patient myoblasts expressing or not expressing dysferlin-GFP (n=11-12), ***p<0.001. (**B**) Representative images of uninjured and post-injury (60s) images of healthy or patient myoblasts mock transfected

or transfected with Dysferlin-GFP. (**C**) Expression of ANO5-GFP and RFP-KDEL in fibrosarcoma cells show ANO5 localizes to the ER.

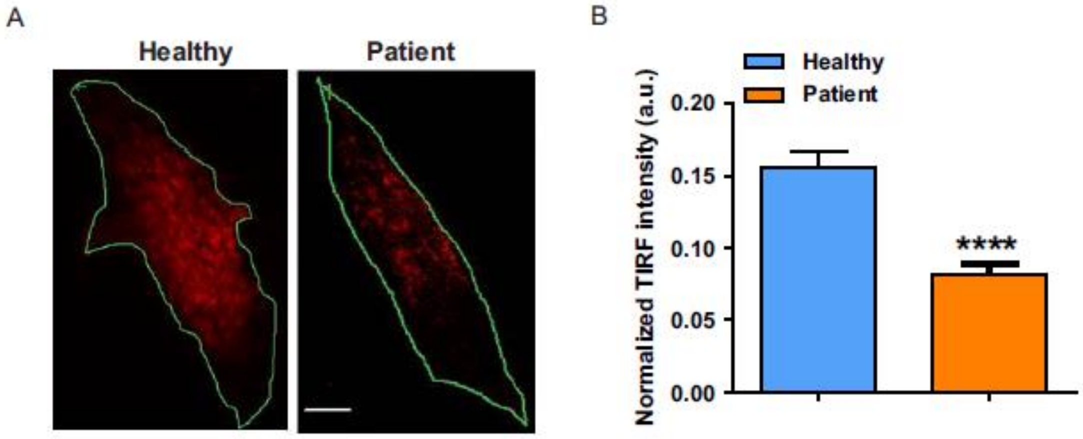


**Supplemental figure 3: Imaging of plasma membrane proximal ER in healthy and patient myoblasts.** (**A**) Images of healthy and patient myoblasts transfected with RFP-KDEL and imaged using TIRF microscopy. (**B**) Ratio quantification of fluorescence intensity of the cells measured simultaneously using TIRF and wide-field microscopy. n = 25 (healthy) and 36 (Patient) from two independent experiments. ****p<0.0001.

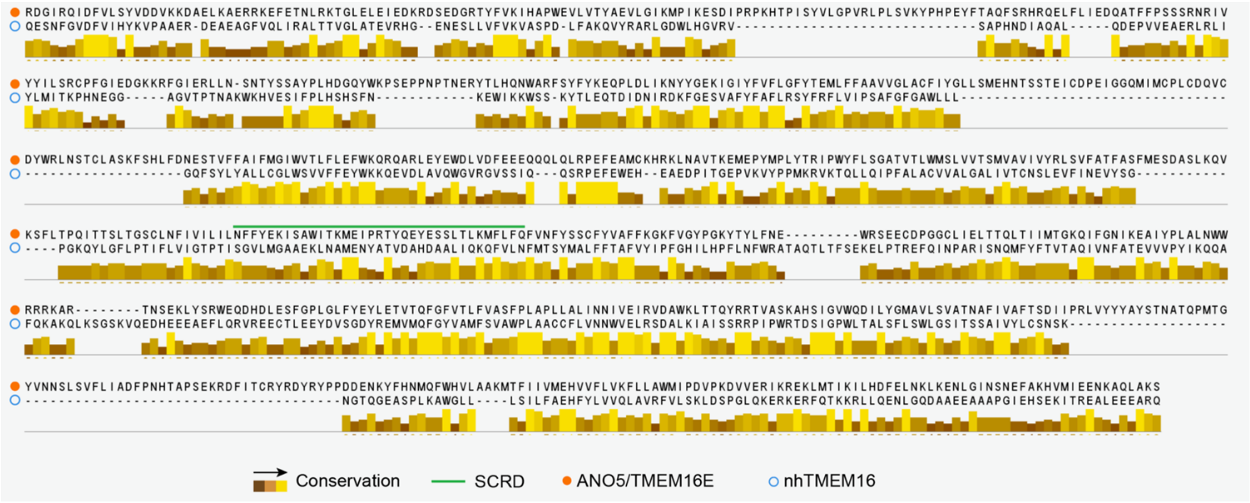


**Supplemental Figure 4: Complete alignment of ANO5 and nhTMEM16 sequences.**

The alignment was generated as described in Materials and Methods, and additional details are the same as described in the Fig 4 legend.

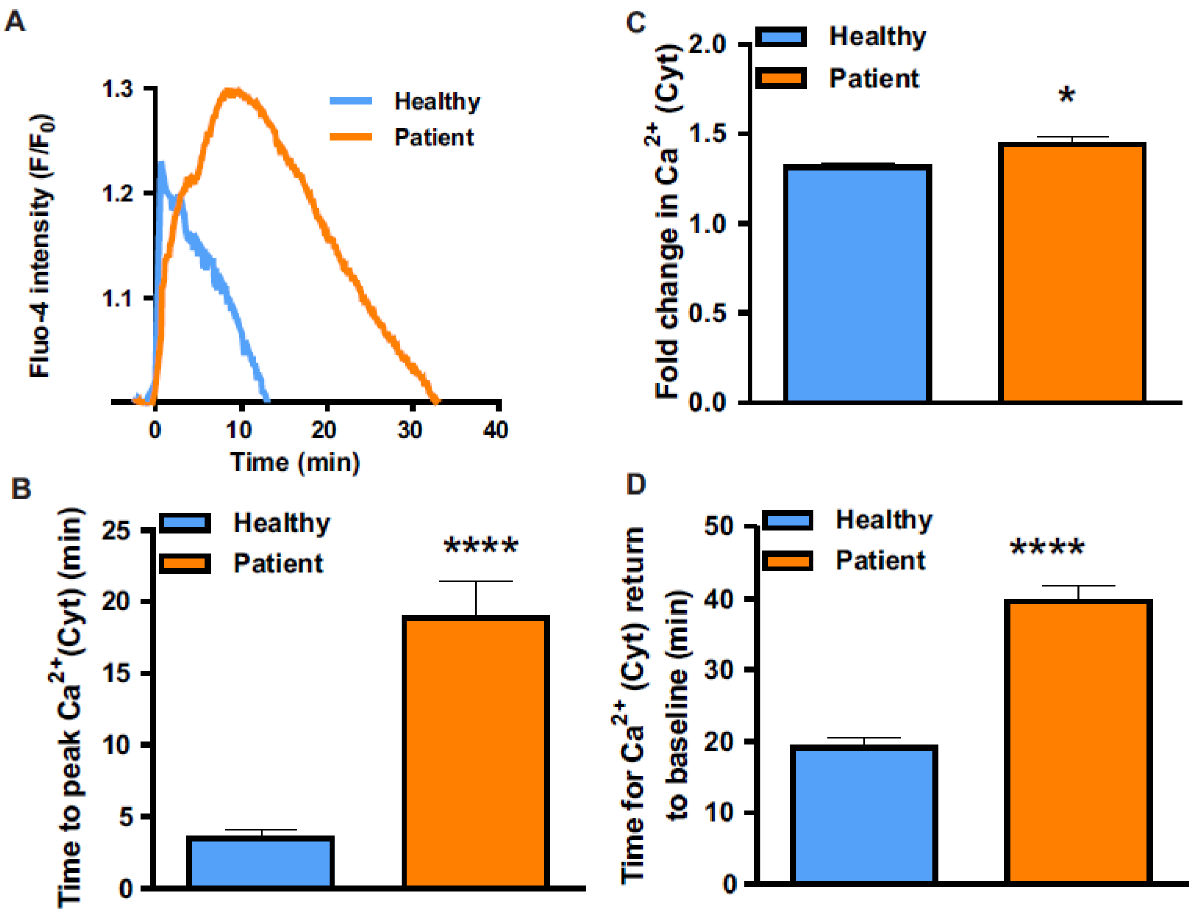


**Supplemental Figure 5: ANO5 deficiency compromises cytosolic $Ca^{2+}$ clearance.** Measurement of cytosolic calcium ($[Ca^{2+}]_{I;}$ by Fluo-4) following ATP stimulation. (**A**) Representative kinetic traces of change in Fluo-4 intensity following ATP treatment. (**B – D**) Quantification of (**B**) The peak increase in $[Ca^{2+}]_c$ following ATP stimulation, (**C**) time taken for the increase in $[Ca^{2+}]_c$ to reach the peak, (*p=0.0294) and (**D**) time taken for the peak $[Ca^{2+}]_c$ to return to pre-stimulation baseline (n=33-40 cells from independent cultures) (****p<0.0001).

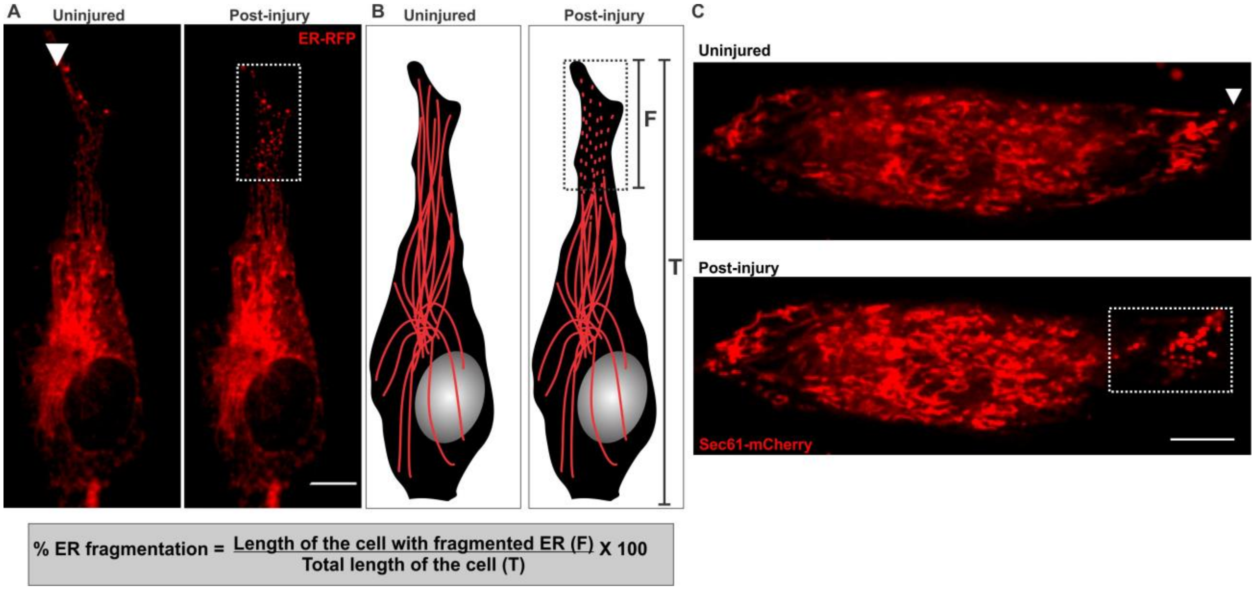


**Supplemental Figure 6: Scheme for calculating extent of ER fragmentation. (A)** Images and (**B**) corresponding schematic of cells transfected with the ER liminal marker ER-RFP and focally injured (arrowhead). White box marks the portion of the cell with fragmented ER. Total length of the cell - “**T**”, and the region of the cell with fragmented ER - “**F**” are marked by capped lines. (**C**) C2C12 myoblast expressing ER membrane marker Sec61-mCherry showing ER fragmentation of at the injury site (arrowhead).

**Legends for supplemental videos:**

**Supplimental video 1**: **Injury-induced ER fragmentation in healthy myoblast**. Video of the healthy human myoblast expressing the ER luminal marker KDEL-RFP and shown in Supplemental figure 6 A, undergoing repair following focal injury (at the top of the frame). . Injury-proximal ER fragments rapidly in response to injury as indicated by the time stamp showin in the second and millisecond format.

**Supplimental video 2**: **Injury-induced ER fragmentation in an ANO5 patient myoblast.** Video of the ANO5 patient (MMD3) human myoblast expressing the ER luminal marker KDEL-RFP showing the extent of ER fragmentation following a focal laser injury (at the top left of the frame). ER fragmentation spreads from the injury proximal site and spreads rapidly throughout the front half of the cell - time stamp shows time in the second and millisecond format.

**Supplimental video 3**: **3D reconstruction showing ER following focal injury of a myoblast.** 3D projection of a C2C12 myoblast expressing the ER membrane marker Sec61-mCherry in Supplemental figure 6 C, after it has repaired from a focal injury (at the top right of the cell). Note the fragmented (beaded) appearance of the injury-proximal ER while the distal ER remains connected.

**Supplemental table 1: Antibodies used in this study**

| Antibody | Catalaogue # | Manufacturer |
|---|---|---|
| Anti-anoctamin 5 (N421A/85) | 73-410 | UC Davis/NIH NeuroMab* |
| Anti-MYH3 (F1.652) | sc-53091 | Santa cruz biotechnologies |
| Anti-desmin | sc-23879 | Santa cruz biotechnologies |
| Anti-α-actinin | sc-17829 | Santa cruz biotechnologies |
| Anti-calnexin | AB2301 | EMD Millipore |
| Anti-GRP78 (76-E6) | sc-13539 | Santa cruz biotechnologies |
| Anti-β-Actin (C-4) HRP | sc-47778 | Santa cruz biotechnologies |

* Validated by – Xu et al 2018[1], Vihola et al 2018[2].

**Supplemental table 2: Plasmids used in this study**

| Plasmid | Source | Reference |
|---|---|---|
| Ano5-GFP | ORFeome Collaboration clone from Open Biosystems (IMAGE ID 100061756) cloned in pEGFP vector – from Dr. Criss Hartzell. | Duran et al., 2012 [3] |
| KDEL-RFP | Addgene plasmid #62236 – Dr. Erik Snapp | Snapp et al., 2006 [4] |
| Sec61-mCherry | Addgene plasmid # 90994 - Dr. Jennifer Lippincott-Schwartz. | Nixon-Abell et al., 2016 [5] |
| Dysferlin-GFP | Dysferlin cDNA derived from plasmid by Dr. Kate Bushby cloned into pEGFP vector. | Klinge et al 2007 [6] ; Defour et al., 2014 [7] |

**References:**

1. Xu, J., *et al.* A novel ANO5 splicing variant in a LGMD2L patient leads to production of a truncated aggregation-prone Ano5 peptide. *J Pathol Clin Res* **4**, 135-145 (2018).
2. Vihola, A., *et al.* Diagnostic anoctamin-5 protein defect in patients with ANO5-mutated muscular dystrophy. *Neuropathol Appl Neurobiol* **44**, 441-448 (2018).

3. Duran, C., Qu, Z., Osunkoya, A.O., Cui, Y. & Hartzell, H.C. ANOs 3-7 in the anoctamin/Tmem16 Cl- channel family are intracellular proteins. *Am J Physiol Cell Physiol* **302**, C482-493 (2012).
4. Snapp, E.L., Sharma, A., Lippincott-Schwartz, J. & Hegde, R.S. Monitoring chaperone engagement of substrates in the endoplasmic reticulum of live cells. *Proc Natl Acad Sci U S A* **103**, 6536-6541 (2006).
5. Nixon-Abell, J.*, et al.* Increased spatiotemporal resolution reveals highly dynamic dense tubular matrices in the peripheral ER. *Science* **354**(2016).
6. Klinge, L.*, et al.* From T-tubule to sarcolemma: damage-induced dysferlin translocation in early myogenesis. *FASEB J* **21**, 1768-1776 (2007).
7. Defour, A.*, et al.* Dysferlin regulates cell membrane repair by facilitating injury-triggered acid sphingomyelinase secretion. *Cell Death Dis* **5**, e1306 (2014).
